# The predictive value of tacrolimus intrapatient variability and time in therapeutic range for renal transplant outcomes

**DOI:** 10.1080/0886022X.2025.2549395

**Published:** 2025-09-03

**Authors:** Ching-Yao Cheng, Hue-Yu Wang, Jaw-Horng Liou, Shang-Feng Tsai

**Affiliations:** ^a^ Department of Pharmacy, Taichung Veterans General Hospital, Taichung City, Taiwan; ^b^ School of Pharmacy, China Medical University, Taichung City, Taiwan; ^c^ Department of Pharmacy, An Nan Hospital, China Medical University, Tainan City, Taiwan; ^d^ Department of Pharmacy, Chia Nan University of Pharmacy and Science, Tainan City, Taiwan; ^e^ Division of Nephrology, Department of Internal Medicine, Taichung Veterans General Hospital, Taichung, Taiwan; ^f^ Department of Life Science, Tunghai University, Taichung, Taiwan; ^g^ Department of Post-Baccalaureate Medicine, College of Medicine, National Chung Hsing University, Taichung, Taiwan; ^h^ Division of Clinical Informatics, Department of Digital Medicine, Taichung Veterans General Hospital, Taichung, Taiwan

**Keywords:** Renal transplantation, tacrolimus, variability, acute rejection, intrapatient variability (IPV), time in therapeutic range (TTR)

## Abstract

**Background:**

Tacrolimus is key in renal transplantation. This study evaluated optimal intrapatient variability (IPV) and time in therapeutic range (TTR) thresholds and association with renal outcomes.

**Methods:**

This single-center study (1999–2018) had a mean follow-up of 3 years. All patients received tacrolimus-based immunosuppression, and excluded those switching from cyclosporin. Receiver operating characteristic curves evaluated IPV and TTR cutoff values for biopsy-proved acute rejection (BPAR). Cox regression, *Chi*-square, Mann–Whitney U, Kaplan–Meier, and log-rank tests were used for outcome analysis.

**Results:**

The cohort included 463 patients (mean age 47.3 years; 44.1% female). Most received cadaveric grafts (66.4%) and ABO-compatible transplants (97.4%). Preexisting diabetes and hypertension were present in 14.7% and 63.7%, respectively. Mean tacrolimus trough level was 7.28 ± 1.92 ng/mL. Patients with IPV ≥25.6% had increased doubling of serum creatinine (DSCr) at 6–12 months (*p* = 0.007) and within 5 years (*p* < 0.001), and higher BPAR within 1 year (*p* = 0.025). Similarly, TTR <81.1% was associated with increased DSCr at 6–12 months (*p* = 0.015) and within 5 years (*p* = 0.009), and higher graft failure rates (*p* = 0.001). An IPV <25.6% was also associated with a lower risk of DSCr within 5 years (HR 0.11; 95% CI, 0.03–0.50) and BPAR within one year (HR 0.59; 95% CI, 0.35–0.98), as shown in the Cox proportional hazards analysis. Kaplan–Meier analysis demonstrated significantly lower survival in patients with IPV ≥25.6% compared to IPV <25.6% (*p* = 0.028), but no significant survival difference between TTR groups (*p* = 0.337).

**Conclusion:**

IPV and TTR are valuable for predicting renal transplant outcomes, with IPV demonstrating stronger predictive ability. Minimizing IPV through frequent monitoring and adherence support could enhance graft survival.

## Introduction

The success of organ transplantation heavily relies on a delicate balance between immunosuppression to prevent graft rejection and avoiding drug toxicity, making therapeutic drug monitoring (TDM) essential [1,2]. Tacrolimus is the cornerstone of the immunosuppressant widely utilized in solid organ transplantation [3], particularly for renal transplants. It is known for its narrow therapeutic index (5–12 ng/mL) [4] and significant intra- and interindividual pharmacokinetic variability [5], which complicates dosing regimens and poses challenges in achieving optimal drug levels in patients post-transplantation [1]. Given the variability in metabolism influenced by genetic factors such as polymorphisms in the CYP3A5 and ABCB1 genes, a growing body of research is focused on developing genotype-based dosing strategies tailored to individual patients [1]. Despite advancements in understanding the pharmacogenetics of tacrolimus, the development of pre-transplant predictive markers for drug levels remains an unmet need in clinical practice [1,6]. Acute rejection (AR) is a significant risk factor for chronic nephropathy and graft loss following renal transplantation, particularly prevalent during the early post-transplant period [7]. Tacrolimus exhibits significant variability in its pharmacokinetics, impacting its efficacy and safety in preventing AR following transplantation. Its trough levels must be carefully monitored, as low concentrations are associated with an increased risk of early acute rejection [2].

In addition to maintaining tacrolimus levels within 5–12 ng/mL after transplantation, the variability in tacrolimus levels also plays a crucial role. The introduction of once-daily formulations of tacrolimus, such as prolonged-release and extended-release versions, aims to enhance patient adherence, simplify dosing regimens, and reduce variability of concentration [8], contributing to fewer tremors [9] and better renal function in some studies [1]. However, the literature still shows inconsistent results regarding the benefits of switching formulations and their impact on renal transplant outcomes, necessitating further investigation [6]. Studies indicate that variability in tacrolimus levels may influence graft survival and adherence among kidney transplant recipients [10,11], suggesting the need for diligent management of immunosuppressant regimens[3]. Studies have highlighted the importance of maintaining stable intrapatient variability (IPV) and time in therapeutic range (TTR) to minimize the risk of AR, particularly in the early post-transplant period [12–14]. However, despite advancements in kidney transplantation, there remains ongoing debate regarding whether high tacrolimus IPV truly impacts graft and/or patient survival. This controversy may arise from variations in the methodologies used to measure IPV (such as standard deviation, coefficient of variation, range, interquartile range, or TTR) and differences in the time frames used for evaluating graft and patient survival in existing studies, including early (0–6 months), mid-term (6–12 months), and long-term (beyond 1 year) periods post-transplantation [12,13,15]. Additionally, to date, limited study has simultaneously investigated tacrolimus variability by focusing on both IPV and TTR within the same cohort in renal transplantation. Therefore, in this study, we aim to investigate the impact of IPV and TTR on renal outcomes across different time frames following renal transplantation.

## Materials and methods

### Definition of population and study design

We enrolled patients who received renal transplantation between January 1, 1999, and December 31, 2018, at this tertiary medical center, including recipients of both living and deceased donor kidneys. Only recipients aged 18 years or older were included. Recipients who underwent more than one renal transplantation or received other organ transplants were excluded. Patients were followed for outcomes through the end of 2018. Since we planned a follow-up period of up to five years, only patients transplanted up to 2018 were included during our 2024 data collection. This study was approved by the Institutional Review Board of Taichung Veterans General Hospital (approval number: CE25001B).

### Methods of data collection

All data were collected from medical records and reviewed by two independent pharmacologists. They recorded all baseline information, including epidemiological data, laboratory results, medical history, and diagnoses, based on the medical records. Outcomes were also documented using pathological and ­laboratory data. All included patients were initiated on tacrolimus within 2 weeks post-transplant and maintained on it throughout. Patients who switched from cyclosporin were excluded.

### Baseline data collection

Data for this study were collected from detailed medical records of renal transplant recipients. Kidney transplant recipient age was recorded in years and categorized into two groups: ≤50 years and >50 years. Biologic sex was documented as male or female, and donor type was classified as either living or cadaveric donors. IPV was calculated using the coefficient of variation (CV) of tacrolimus trough levels during the 6–12 months post-transplantation. TTR was assessed using the Rosendaal method (linear interpolation) and recorded for two periods: within 6–12 months and within 1 year post-transplantation. Preexisting comorbidities, including diabetes mellitus, hypertension, hepatitis C virus infection, and hepatitis B virus infection, were documented based on the patients’ medical history. Results from two panel reactive antibody (PRA) tests class I and II were recorded, along with the number of human leukocyte antigens (HLA) mismatches between donor and recipient. ABO incompatibility between donor and recipient was documented, and the use of interleukin-2 receptor antibody for induction therapy was recorded as a binary variable (yes or no). Other immunosuppressive medications, including prednisolone and preparations of mycophenolic acid: mycophenolate mofetil (CellCept), and enteric-coated mycophenolate sodium (Myfortic), were also recorded. All data were reviewed and verified by two independent pharmacologists to ensure accuracy and consistency.

### Pre-specified outcome defintion

Doubling of serum creatinine (DSCr) was recorded at two key intervals: within 6–12 months (mid-term outcome) and within 5 years post-transplantation (long-term outcome). We defined doubling as an increase to ≥2× baseline at any time during follow-up. Transient fluctuations were considered; a sustained doubling for >1 month was required to meet the endpoint. DSCr post-transplant has been established as a surrogate endpoint, supported by its association with histopathological evidence from renal allograft biopsies [16].

Biopsy-proven acute rejection (BPAR) was defined as acute rejection episodes confirmed by biopsy and recorded within 1 year (mid-term) and 6 months (short-term) after transplantation. BPAR was defined according to Banff 2013 classification [17]. Mortality was defined as death from any cause during the follow-up period, while graft failure was defined as the requirement for dialysis or repeat transplantation.

Receiver operating characteristic (ROC) curves for IPV and TTR were used to assess their diagnostic accuracy in predicting DSCr within five years post-transplantation (long-term outcome). The identified cutoff values for IPV and TTR were then applied to categorize patients accordingly. Cox proportional hazards analysis was conducted to evaluate risk factors associated with DSCr within five years and biopsy-proven acute rejection (BPAR) within one year. Kaplan–Meier survival curves were generated to assess BPAR within the first year post-transplantation (mid-term outcome).

### The definition of variability in tacrolimus trough levels: IPV and TTR

At our institution, tacrolimus trough levels were adjusted in accordance with the KDIGO 2009 [3] and 2020 guidelines [18]. The target range (5–12 ng/mL) was consistent with local clinical practice, with dynamic modifications based on the post-transplantation phase. IPV was defined based on all tacrolimus trough values recorded from 6 to 12 months post-transplantation (excluding outlier, > 20 ng/mL) [19]. Otherwise, values >20 ng/mL with uncertain timing were considered potential non-trough samples and excluded to reduce bias. IPV was calculated using the CV [20–22], defined as: coefficient of variation: CV (%) = (standard deviation/mean) * 100.

In addition to IPV, TTR was used as another measure of tacrolimus level variability. The TTR was calculated using the Rosendaal Method (Linear Interpolation) [23], which estimates the proportion of time (from 6 to 12 months post-transplantation) that tacrolimus concentrations remained within the target therapeutic range. To determine the cutoff value of TTR, we also evaluated another time point (1 year post-transplantation) to predict a DSCr within 5 years after transplantation. In this study, we established a target therapeutic range for tacrolimus of 5–12 ng/mL.

### Tacrolimus measurements

Tacrolimus whole blood concentrations were measured using a chemiluminescent microparticle immunoassay (CMIA) on the ARCHITECT i2000SR system (Abbott Diagnostics, Abbott Park, IL, USA) [24,25]. This assay employs paramagnetic microparticles coated with tacrolimus-specific monoclonal antibodies to selectively bind tacrolimus molecules in EDTA-treated whole blood samples. Prior to analysis, whole blood samples were treated with a proprietary pretreatment solution to lyse red blood cells and release tacrolimus from erythrocytes, where it is primarily distributed. Following this, tacrolimus competes with a tacrolimus–acridinium-labeled conjugate for binding to the antibody-coated microparticles. After a wash step to remove unbound material, a chemiluminescent reaction is initiated. The resulting relative light units (RLUs) emitted are inversely proportional to the concentration of tacrolimus in the sample.

All measurements were performed within 24 h of sample collection to ensure analyte stability. Quality control procedures, including two levels of internal control material, were run with each analytical batch to ensure consistency and accuracy.

Tacrolimus trough concentrations were measured 2–3 times weekly during the first month, weekly during months 2–3, biweekly until 6 months, and monthly thereafter as per our center’s protocol.

## Sensitivity analysis

Sensitivity analyses were performed to evaluate the impact of previously published thresholds [26–29]—IPV <30% and TTR ≥75%—on renal outcomes in this cohort, using Cox proportional hazards analysis and Kaplan–Meier survival curves.

### Statistical analyses

Statistical analyses were conducted using appropriate methods to evaluate the relationship between tacrolimus IPV, TTR, and post-transplant outcomes. The *Chi*-square test was employed for categorical variables, while the Mann-Whitney U test was used for continuous variables with non-normal distributions. ROC curves were utilized to assess the performance metric for cutoff discrimination of IPV and TTR for acute rejection, with the area under the curve (AUC) providing a measure of discriminative ability. Cox proportional hazards analysis was performed to evaluate the risk factors associated with the outcome. Kaplan–Meier survival analysis, accompanied by the log-rank test, was performed to compare survival rates between groups. All analyses were conducted with a significance level set at *p* < 0.05. All analyses were conducted using SPSS statistical software, version 12.0 (SPSS Inc., Chicago, IL, USA).

## Results

### Baseline status of the whole cohort

The patient selection algorithm is presented in Supplementary Figure 1. After applying specific selection criteria, we identified 463 patients for further analysis. The study cohort included 463 patients, with a mean age of 47.3 years, of whom 44.1% were female (Table 1). The majority of patients (66.4%) received transplants from cadaveric donors, and 97.4% of the transplants were ABO-compatible. Preexisting diabetes mellitus was present in only 14.7% of the recipients, while 63.7% had hypertension prior to transplantation. The primary cause of uremia is unknown in 31% of cases. The mean trough level of tacrolimus is 7.28 ± 1.92 ng/mL. Tacrolimus trough levels were measured a median of 15 times within the first 6 months and 23 times within the first year post-transplantation.

**Table 1.** Baseline characteristics of patients with IPV ≥25.6% or <25.6%.

	Total		IPV < 25.6%		IPV ≥ 25.6%		*p* value
Patient number	463		259		204		
Kidney Transplant Recipient Age (years) (mean)	46.2	±12.2	45.6	±12.1	46.9	±12.2	0.276
**Recipient Sex **							**0.438**
Female	204	(44.1%)	110	(42.5%)	94	(46.1%)	
Male	259	(55.9%)	149	(57.5%)	110	(53.9%)	
**Cause of ESKD **							
Primary glomerulonephritis	126	27%	73	28%	53	26%	0.253
Diabetes mellitus	55	12%	30	12%	25	12%	0.631
Hypertension	33	7%	18	7%	15	7%	0.135
Autosomal dominant polycystic kidney disease	23	5%	15	6%	8	4%	0.746
Lupus nephritis	22	5%	15	6%	7	3%	0.746
Non-steroid analgesics related	55	12%	31	12%	24	12%	0.469
Obstructive uropathy	5	1%	4	2%	1	0%	0.463
Uknown	144	31%	73	28%	71	35%	0.146
**Donor type **							0.036*
Living donor	165	(35.6%)	103	(39.8%)	62	(30.4%)	
Cadaveric donor	298	(64.4%)	156	(60.2%)	142	(69.6%)	
**Immune suppressive medical therapy **							
**Mycophenolate mofetil**	1500	(1500–2000)	1500	(1500–2000)	1500	(1000–2000)	0.170
Prednisolone	30	(30–30)	30	(30–30)	30	(30–30)	0.847
**Mycophenolate sodium**	1080	(720–1440)	1080	(720–1440)	1080	(720–1440)	0.944
**Times of tacrolimus measurement **							
Tacrolimus frequency within 6 months	15	(13–19)	15	(13–18)	15	(12–20)	0.617
Tacrolimus frequency within 1 year	23	(20–28)	23	(19–26)	23	(20–30)	0.005**
Mean trough level of tacroliumus (ng/mL)	7.28	1.92	7.20	1.65	7.39	2.23	0.408
Median trough level of tacroliumus (ng/mL) (IQR)	7.2	(6.1–8.4)	7.1	(6.2–8.3)	7.3	(5.7–8.6)	0.618
IPV	23.9%	(16.9%–33.7%)	17.5%	(13.7%–21.2%)	35.0%	(30.2%–46.0%)	<0.001**
TTR within 6–12 months after transplantation	88.0%	(63.4%–100.0%)	98.6%	(81.0%–100.0%)	74.5%	(51.8%–89.6%)	<0.001**
TTR within 1 year after transplantation	81.9%	(65.2%–92.2%)	88.0%	(76.0%–94.2%)	71.4%	(57.1%–85.4%)	<0.001**
**Comorbidity **							
Preexisting Diabetes Mellitus	62	(14.7%)	31	(12.8%)	31	(17.1%)	0.214
Preexisting Hypertension	295	(63.7%)	179	(69.1%)	116	(56.9%)	0.006**
Preexisting Hepatitis C Virus	20	(4.5%)	9	(3.6%)	11	(5.7%)	0.286
Preexisting Hepatitis B Virus	38	(8.6%)	22	(8.8%)	16	(8.2%)	0.826
Panel Reactive Antibody Test 1	42	(9.1%)	20	(7.8%)	22	(10.8%)	0.266
Panel Reactive Antibody Test 2	27	(5.9%)	12	(4.7%)	15	(7.4%)	0.223
Number of HLA Mismatches	1.0	(0.0–2.0)	1.0	(0.0–3.0)	1.0	(0.0–2.0)	0.029*
ABO Incompatibility	12	(2.6%)	6	(2.3%)	6	(2.9%)	0.675
Use of Interleukin-2 Receptor Antibody for Induction	155	(34.6%)	97	(38.6%)	58	(29.4%)	0.042*
**Outcome **							
Doubling of Serum Creatinine within 6–12 months	50	(10.8%)	19	(7.3%)	31	(15.2%)	0.007**
Doubling of Serum Creatinine within 5 years	20	(4.3%)	2	(0.8%)	18	(8.8%)	<0.001**
Mean duration of follow-up (years)	2.7	±1.5	1.5	±2.1	2.8	±1.4	
Biopsy-Proven Acute Rejection within 1 year	61	(13.2%)	26	(10.0%)	35	(17.2%)	0.025*
Mean duration of follow-up (years)	0.2	±0.3	0.2	±0.3	0.3	±0.3	
Biopsy-Proven Acute Rejection within 6 months after transplantation	13	(2.8%)	5	(1.9%)	8	(3.9%)	0.198
Mortality	65	(14.0%)	33	(12.7%)	32	(15.7%)	0.365
Mortality (within 5 year)	20	(4.3%)	11	(4.2%)	9	(4.4%)	0.931
Mean duration of follow-up (years)	3.1	±1.3	3.2	±1.3	3.1	±1.4	
Graft failure	97	(21.0%)	46	(17.8%)	51	(25.0%)	0.057
Graft failure (5 years)	34	(7.3%)	17	(6.6%)	17	(8.3%)	0.469
Mean duration of follow-up (years)	3.2	±1.3	3.2	±1.1	3.1	±1.5	

IPV: Intrapatient Variability.

*Chi*-square test or Mann–Whitney *U* test, Median (IQR). *p < 0.05, **p < 0.01.

### Baseline status of the whole cohort stratified by IPV < 25.6% or IPV ≥ 25.6%

**Figure 1.**
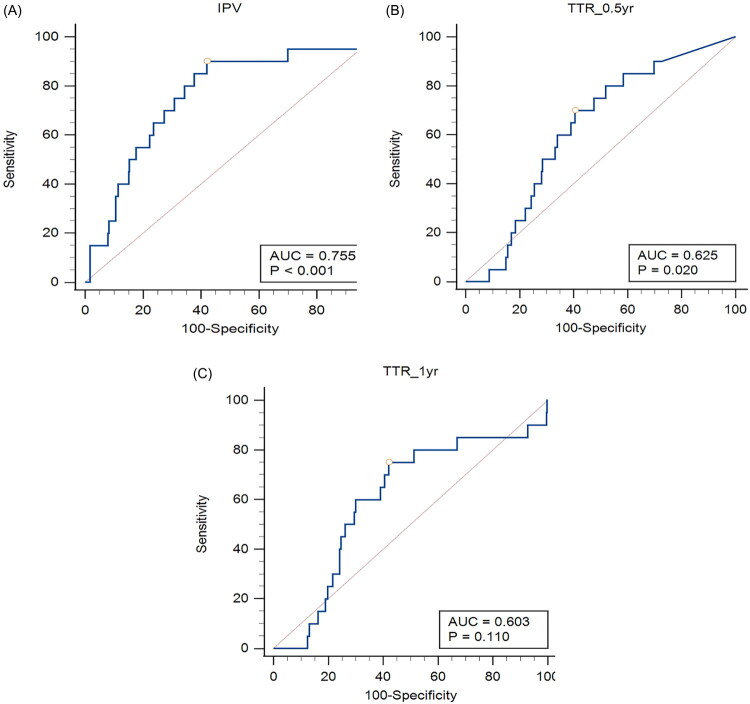
(A) ROC curve for predicting doubling of serum creatinine within 5 years after transplantation using IPV (IPV ≥ 25.6, sensitivity = 90%, specificity = 58.0%). (B) ROC Curve for predicting doubling of serum creatinine within 5 Years after Transplantation Using TTR (TTR with 6–12 months) (TTR < 81.1%, sensitivity = 70%, specificity = 58.4%). (C) ROC Curve for predicting doubling of serum creatinine within 5 years after transplantation using TTR (TTR within 1 year) (TTR < 79.0%, sensitivity = 75.0%, specificity = 58.0%).

As shown in Figure 1(A), the ROC curve for predicting DSCr within 5 years post-transplantation identified a cutoff at IPV ≥ 25.6%, with a sensitivity of 90% and specificity of 58.0%. The performance metric for cutoff discrimination was acceptable, with an AUC of 0.775 and *p* < 0.001. Consequently, the cohort was divided into two groups based on IPV: IPV ≥ 25.6% and IPV < 25.6% (Table 1). Patients in the high IPV group (IPV ≥ 25.6%) were more likely to have received a transplant from a cadaveric donor (69.6% vs. 60.2%, *p* = 0.036). They also exhibited significantly lower TTR at both 6-12 months (74.5% vs. 98.6%, *p* < 0.001) and 1 year (71.4% vs. 88.0%, *p* < 0.001) post-transplantation. Additionally, the high IPV group had fewer cases of preexisting hypertension (56.9% vs. 69.1%, *p* = 0.006), more HLA mismatches (*p* = 0.029), and lower induction rates of Interleukin-2 Receptor Antibody (29.4% vs. 38.6%, *p* = 0.042).

### Baseline status of the whole cohort stratified by TTR < 81.1% or TTR ≥ 81.1%

In Figure 1(C), TTR < 81.1% was identified as a predictor for an increased risk of SCR within 5 years post-transplantation, with an AUC of 0.625, sensitivity of 70%, and specificity of 58.4% (*p* = 0.020). Accordingly, the cohort was divided into two groups based on TTR: TTR < 81.1% and TTR ≥ 81.1% (Table 2). Patients with TTR < 81.1% were significantly more likely to have received a transplant from a cadaveric donor (71.6% vs. 59.1%, *p* = 0.005) and exhibited higher IPV (32.1% vs. 20.1%, *p* < 0.001).

**Table 2.** Baseline characteristics of patients with TTR ≥81.1% or <81.1%.

	Total		TTR < 81.1%		TTR ≥ 81.1%		*p* value
Patient number	463		194		269		
Kidney Transplant Recipient Age (years) (mean)	46.2	±12.2	46.9	±11.2	45.6	±12.8	0.248
**Sex **							0.504
Female	204	(44.1%)	89	(45.9%)	115	(42.8%)	
Male	259	(55.9%)	105	(54.1%)	154	(57.2%)	
**Cause of ESKD **							
Primary glomerulonephritis	126	27%	45	23%	81	30%	0.123
Diabetes mellitus	55	12%	29	15%	26	10%	0.236
Hypertension	33	7%	17	9%	16	6%	0.536
Autosomal dominant polycystic kidney disease	23	5%	6	3%	17	6%	0.463
Lupus nephritis	22	5%	5	3%	17	6%	0.496
Non-steroid analgesicsrelated	55	12%	26	13%	29	11%	0.963
Obstructive uropathy	5	1%	1	1%	4	1%	0.636
Unknown	144	31%	65	34%	79	30%	0.132
Donor type							0.005**
Living donor	165	(35.6%)	55	(28.4%)	110	(40.9%)	
Cadaveric donor	298	(64.4%)	139	(71.6%)	159	(59.1%)	
**Immune suppressive medical therapy**							
**Mycophenolate mofetil**	1500	(1500–2000)	1500	(1000–2000)	1500	(1500–2000)	0.034*
Prednisolone	30	(30–30)	30	(30–30)	30	(30–30)	0.592
**Mycophenolate sodium**	1080	(720–1440)	1080	(720–1440)	1080	(720–1440)	0.160
**Times of tacrolimus measurement **							
Tacrolimus frequency within 6 months	15	(13–19)	16	(12–19)	15	(13–18)	0.456
Tacrolimus frequency within 1 year	23	(20–28)	24	(20–29.5)	23	(20–27)	0.102
Mean trough level of tacroliumus (ng/mL)	7.28	1.92	6.80	2.60	7.60	1.18	<0.001**
Median trough level of tacroliumus (ng/mL) (IQR)	7.2	(6.1–8.4)	6.0	(4.9–8.3)	7.4	(6.6–8.4)	<0.001**
IPV	23.9%	(16.9%–33.7%)	32.1%	(22.0%–45.7%)	20.1%	(15.2%–26.7%)	<0.001**
TTR within 6–12 months after transplantation	88.0%	(63.4%–100.0%)	57.5%	(29.8%–72.5%)	99.1%	(91.8%–100.0%)	<0.001**
TTR within 1 year after transplantation	81.9%	(65.2%–92.2%)	62.1%	(44.6%–75.9%)	90.1%	(82.4%–94.7%)	<0.001**
Comorbidity							
Preexisting Diabetes Mellitus	62	(14.7%)	31	(17.8%)	31	(12.4%)	0.125
Preexisting Hypertension	295	(63.7%)	115	(59.3%)	180	(66.9%)	0.092
Preexisting Hepatitis C Virus	20	(4.5%)	9	(4.9%)	11	(4.3%)	0.770
Preexisting Hepatitis B Virus	38	(8.6%)	16	(8.6%)	22	(8.6%)	0.988
Panel Reactive Antibody Test 1	42	(9.1%)	23	(11.9%)	19	(7.1%)	0.081
Panel Reactive Antibody Test 2	27	(5.9%)	13	(6.7%)	14	(5.2%)	0.511
Number of HLA Mismatches	1	(0.0–2.0)	0.0	(0.0–2.0)	1.0	(0.0–2.0)	0.540
ABO Incompatibility	12	(2.6%)	4	(2.1%)	8	(3.0%)	0.542
Use of Interleukin-2 Receptor Antibody for induction	155	(34.6%)	62	(33.0%)	93	(35.8%)	0.540
**Outcome **							
Doubling of Serum Creatinine within 6–12 months	50	(10.8%)	29	(14.9%)	21	(7.8%)	0.015*
Doubling of Serum Creatinine within 5 years	20	(4.3%)	14	(7.2%)	6	(2.2%)	0.009**
Mean duration of follow-up (years)	2.7	±1.5	2.7	±1.6	2.6	±1.4	
Biopsy-Proven Acute Rejection within 1 year	61	(13.2%)	29	(14.9%)	32	(11.9%)	0.338
Mean duration of follow-up (years)	0.2	±0.3	0.3	±0.3	0.2	±0.3	
Biopsy-Proven Acute Rejection within 6 months after transplantation	13	(2.8%)	6	(3.1%)	7	(2.6%)	0.753
Mortality	65	(14.0%)	30	(15.5%)	35	(13.0%)	0.454
Mortality (within 5 year)	20	(4.3%)	9	(4.6%)	11	(4.1%)	0.774
Mean duration of follow-up (years)	3.1	±1.3	3.1	±1.4	3.2	±1.3	
Graft failure	97	(21.0%)	55	(28.4%)	42	(15.6%)	0.001**
Graft failure (within 5 years)	34	(7.3%)	19	(9.8%)	15	(5.6%)	
Mean duration of follow-up (years)	3.2	±1.3	3.2	±1.5	3.2	±1.0	

*Chi*-square test or Mann–Whitney U test, Median (IQR). *p < 0.05, **p < 0.01.

#### Renal outcomes between IPV < 25.6% or IPV ≥ 25.6%

In Table 1, high IPV groups experienced more instances of DSCr at 6-12 months (15.2% vs. 7.3%, *p* = 0.007) and at 5 years (8.8% vs. 0.8%, *p* < 0.001) post-transplantation. Additionally, the high IPV group had a higher rate of BPAR within 1 year (17.2% vs. 10.0%, *p* = 0.025). They also exhibited a numerically higher mortality rate (25.0% vs. 17.8%, *p* = 0.057).

**Figure 2.**
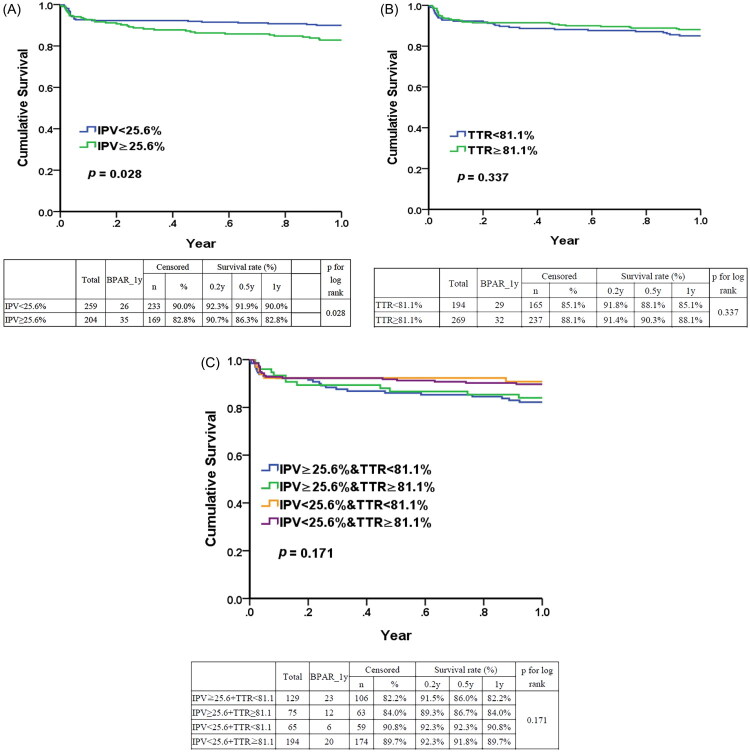
Kaplan–Meier Survival curve of biopsy-proven acute rejection within 1 year post-renal transplantation. (A) Between IPV < 25.6% and ≥25.6%. (B) Between TTR< 81.1% and TTR ≥ 81.1. (C) Among four groups based on IPV (≥25.6% or <25.6%) and TTR (≥81.1% or <81.1%).

The low IPV group was associated with a lower risk of DSCr at 5 years (HR 0.11; 95% CI, 0.03–0.50) compared to the high IPV group (Table 3). The high IPV group showed strong predictive power for DSCr at 5 years, with an AUC of 0.755 (*p* < 0.001) (Figure 1(A)). Similarly, the low IPV group had a reduced risk of BPAR (HR 0.59; 95% CI, 0.35–0.98) compared to the high IPV group (Table 4). The high IPV group also exhibited a significantly higher risk of BPAR within one year post-transplantation (*p* = 0.028) (Figure 2(A)).

**Table 3.** Cox proportional hazard analysis for doubling of serum creatinine (within 5 years).

	Univariate			Multivariable		
	Hazard ratio	95%CI	*p* value	Hazard ratio	95%CI	*p* value
Sex						
** **Female	Reference					
** **Male	0.52	(0.21–1.28)	0.157			
Age	0.95	(0.92–0.99)	0.013*	0.94	(0.89–0.98)	0.005**
Donor type						
** **Living	Reference					
** **Cadaveric	1.29	(0.50–3.35)	0.603			
Preexisting disease hypertension	2.34	(0.78–7.00)	0.129			
** **Diabetes mellitus	1.48	(0.50–4.43)	0.482			
Preformed antibody class I	0.51	(0.07–3.83)	0.516			
Preformed antibody class II	4.38	(1.46–13.11)	0.008**	20.30	(3.75–109.82)	<0.001**
Number of HLA mismatch	0.89	(0.66–1.20)	0.435			
Cause of end-stage kidney disease						
Primary glomerular disease	Reference			Reference		
** **Diabetes mellitus	1.27	(0.42–3.78)	0.672	2.26	(0.63–8.17)	0.212
** **Hypertension	0.84	(0.18–3.90)	0.827	1.04	(0.21–5.17)	0.962
** **NSAID or herbs	0.24	(0.03–1.91)	0.178	0.29	(0.03–2.59)	0.265
** **Unknow cause	0.22	(0.03–1.77)	0.157	0.18	(0.02–1.51)	0.114
Obstructive uropathy, ADPKD, and lupus nephritis	0.20	(0.04–0.93)	0.041*	0.07	(0.01–0.52)	0.009**
Use of Interleukin-2 Receptor Antibody for induction	0.33	(0.10–1.13)	0.077	0.11	(0.02–0.60)	0.010*
IPV						
** **IPV ≥ 25.6%	Reference			Reference		
** **IPV < 25.6%	0.08	(0.02–0.36)	0.001**	0.11	(0.03–0.50)	0.004**
TTR						
** **TTR < 81.1%	Reference			Reference		
** **TTR ≥ 81.1%	0.30	(0.12–0.79)	0.014*	0.42	(0.15–1.20)	0.104

HLA: human leukocyte antigen; NSAID: non-steroidal anti-inflammatory drugs; ADPKD: Autosomal dominant polycystic kidney disease; IPV: intra-patient variability; TTR: time in target range.

Cox proportional hazard regression. *p < 0.05, **p < 0.01.

**Table 4.** Cox proportional hazard analysis for r biopsy-proven acute rejection (within 1 year).

	Univariate			Multivariable		
	Hazard ratio	95%CI	*p* value	Hazard ratio	95%CI	*p* value
Sex						
** **Female	Reference					
** **Male	1.07	(0.64–1.77)	0.802			
Age	1.01	(0.99–1.03)	0.437			
Donor type						
** **Living	Reference			Reference		
** **Cadaveric	0.55	(0.33–0.91)	0.020*	0.57	(0.34–0.96)	0.034*
Preexisting disease hypertension	1.63	(0.92–2.89)	0.092			
Diabetes mellitus	1.65	(0.87–3.14)	0.123			
Preformed antibody class I	2.13	(1.08–4.20)	0.029*	2.30	(1.14–4.67)	0.021*
Preformed antibody class II	2.29	(1.04–5.02)	0.040*			
Number of HLA mismatch	1.03	(0.88–1.19)	0.745			
Cause of end-stage kidney disease						
Primary glomerular disease	Reference					
** **Diabetes mellitus	1.48	(0.67–3.26)	0.331			
** **Hypertension	1.53	(0.60–3.90)	0.378			
** **NSAID or herb	1.37	(0.61–3.10)	0.451			
** **Unknow cause	1.21	(0.54–2.74)	0.643			
Obstructive uropathy, ADPKD, and lupus nephritis	0.65	(0.30– 1.39)	0.264			
Use of Interleukin-2 Receptor Antibody for induction	0.50	(0.27–0.92)	0.026*	0.44	(0.23–0.82)	0.010*
IPV						
** **IPV ≥ 25.6%	Reference			Reference		
** **IPV < 25.6%	0.57	(0.34–0.95)	0.031*	0.59	(0.35–0.98)	0.040*
TTR						
** **TTR < 81.1%	Reference					
** **TTR ≥ 81.1%	0.78	(0.47–1.29)	0.339			

HLA: human leukocyte antigen; NSAID: non-steroidal anti-inflammatory drugs; ADPKD: Autosomal dominant polycystic kidney disease; IPV: intra-patient variability; TTR: time in target range.

Cox proportional hazard regression. *p < 0.05

#### Renal outcomes between TTR < 81.1% or TTR ≥ 81.1%

In Table 2, the low TTR group exhibited a higher incidence of DSCr at 6-12 months (14.9% vs. 7.8%, *p* = 0.015) and at 5 years (7.2% vs. 2.2%, *p* = 0.009) post-transplantation. They also had a significantly higher risk of graft failure (28.4% vs. 15.6%, *p* = 0.001).

The low TTR group (6–12 months) (<81.1%) can predict a DSCr 5 years after transplantation (AUC = 0.625, *p* = 0.020) (Figure 1(B)). In contrast, the TTR (1–12 months) (<79%) cannot predict renal outcomes (Figure 1(C)). Additionally, the low and high TTR groups (6–12 months) (81.1%) showed similar survival outcomes for BPAR within 1 year (Figure 2(B)). Stratification by TTR did not improve the performance metric for cutoff discrimination of IPV (<25.6% or ≥25.6%) for BPAR (Figure 2(C) and supplementary Table 1).

#### Sensitivity analysis

**Figure 3.**
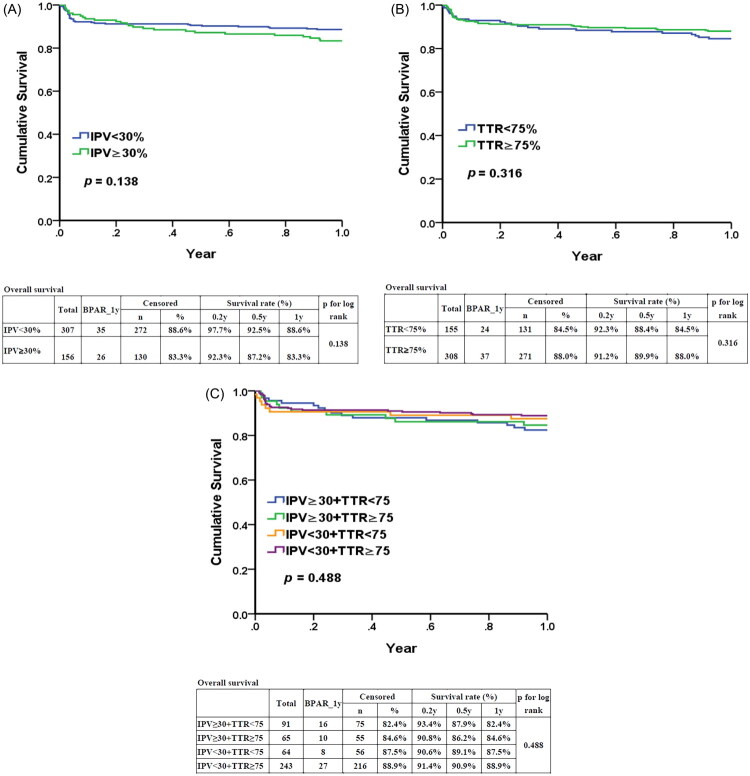
Sensitivity analysis of different cutoff-value of IPV < 30% and TTR ≥ 75%. (A) Between IPV < 30% and ≥30%; (B) Between TTR < 75% and TTR ≥75; (C) Among four groups based on IPV (≥30% or <30%) and TTR (≥75% or <75%).

BPAR within one year in patients with IPV <30% and TTR ≥75% The use of published thresholds for IPV and TTR (IPV <30% and TTR ≥75%) is illustrated in Figure 3. Neither IPV <30% (Figure 3(A)), TTR ≥75% (Figure 3(B)), nor the combined condition of IPV <30% and TTR ≥75% (Figure 3(C)) showed any significant difference in BPAR-free survival within one year.

## Discussion

This is the first study to simultaneously investigate both IPV and TTR in the same cohort at different time periods for renal outcomes. This study highlights the importance of IPV and TTR in predicting mid-term and long-term renal outcomes following kidney transplantation.

Our findings suggest that high IPV (IPV ≥ 25.6%) is a significant predictor of adverse renal outcomes, including a higher incidence of DSCr and BPAR. The high IPV group exhibited a significantly higher risk of DSCr at both 6-12 months (mid-term) (15.2% vs. 7.3%, *p* = 0.007) and 5 years (long-term) post-transplantation (8.8% vs. 0.8%, *p* < 0.001). Furthermore, this group showed a higher rate of BPAR within the first year (17.2% vs. 10.0%, *p* = 0.025) and a numerically higher graft failure rate (25.0% vs. 17.8%, *p* = 0.057) (long-term). Importantly, the performance metric for cutoff discrimination of high IPV for DSCr at 5 years was robust, with an AUC of 0.755 (*p* < 0.001). This cutoff value aligns with findings from another study published in 2023 [30], where IPV > 26.5% was associated with calcineurin inhibitor-related toxicity and poorer 5-year graft survival. Similarly, Borra et al. also publish a study in 2010 [11], using a similar cutoff value of IPV (24.2%). High IPV patients had worse composite renal outcome (graft failure, chronic allograft nephropathy, or doubling of serum creatinine). Another study found that IPV > 30% was independently associated with death-censored graft loss (HR = 2.613, 95% CI: 1.361–5.016) and the development of donor-specific antibody (HR = 2.925, 95% CI: 1.473–5.807) [10]. These results highlight the clinical utility of IPV as an early indicator for identifying patients at risk of graft dysfunction, including both mid-term and long-term outcomes, supported by laboratory and pathological evidence.

In addition to IPV, patients with low TTR demonstrated a higher incidence of DSCr at both 6-12 months (14.9% vs. 7.8%, *p* = 0.015) (mid-term) and 5 years (7.2% vs. 2.2%, *p* = 0.009) (long-term) post-transplantation. Furthermore, these patients had a markedly higher risk of graft failure (28.4% vs. 15.6%, *p* = 0.001). The cutoff value is consistent with another study [31], in which TTR < 78% was associated with graft loss (0.733 of AUC). In our study, despite its lower performance metric for cutoff discrimination compared to IPV (AUC = 0.625, *p* = 0.020), TTR remains an important parameter to monitor in clinical practice, particularly during the early post-transplantation period.

In this study, low IPV corresponded to high TTR, and both IPV and TTR were associated with adverse renal outcomes. This strong correlation has also been reported in a previous study [1]. However, IPV is not equivalent to TTR. When comparing these two markers, IPV showed a stronger performance metric for cutoff discrimination for long-term outcomes, including DSCr and BPAR. Stratification by TTR did not improve the performance metric for cutoff discrimination of IPV for BPAR (Figure 2(C) and Supplementary Table 1), suggesting that IPV may be a more reliable standalone metric in clinical decision-making. Additionally, although the low and high TTR groups demonstrated similar survival outcomes for BPAR within 1 year (Figure 2(B)), IPV was more closely associated with both BPAR and graft survival, highlighting its superior prognostic value. These results contrast with those of another study [31], which reported that tacrolimus TTR was more strongly associated with patient and graft survival than IPV, suggesting that TTR may be a more reliable indicator for tacrolimus monitoring. That study employed a narrower target range (5–10 ng/mL during months 0–3 and 4–8 ng/mL during months 4–12) compared to our study (5–12 ng/mL during months 6–12). In contrast, another study [32] found no significant association between tacrolimus TTR and the risk of BPAR. These discrepancies highlight that the predictive value of TTR or IPV is heavily influenced by the methodology used to calculate variability, the frequency of trough level measurements, and the definition of target ranges. From a pharmacological perspective, TTR may offer a more clinically meaningful measure than IPV. For example, a patient with consistently undetectable tacrolimus levels may exhibit low (seemingly acceptable) IPV, yet their TTR would be unacceptably low—indicating underexposure. Conversely, a patient with persistently supratherapeutic levels may also show low IPV but have a dangerously high TTR. Therefore, we believe the key issue is not which metric is inherently superior, but rather which measure provides greater clinical utility in specific clinical contexts.

Theoretically, there are many differences between IPV and TTR. Both parameters are important for assessing drug therapy, but they serve different purposes: IPV focuses on variability [19], while TTR focuses on stability within the therapeutic range [33,34]. A high TTR can indicate good control, but it does not capture variability between measurements. Patients with high TTR but high IPV may still be at risk of graft dysfunction due to fluctuating tacrolimus levels. Conversely, patients with low TTR but low IPV may have more stable exposure and better outcomes if their levels remain near the edges of the therapeutic range without large swings. In addition, TTR is closely associated with achieving the therapeutic target range, which should be adjusted based on different immunological risks, including HLA mismatch, ABO compatibility, and crossmatch status. The appropriate TTR cutoff varies based on different conditions. As a result, TTR is not as straightforward as IPV. Thirdly, while IPV is relatively simple to calculate, determining TTR is more complex. In summary, for tacrolimus use in renal transplantation, IPV is generally more critical than TTR, as it offers a more accurate reflection of a patient’s risk of graft dysfunction and rejection. We believe that the differing performance metric for cutoff discrimination of IPV and TTR was due to the pre-defined therapeutic range for TTR, set at 5–12 ng/mL in this study. If the range were narrowed to 5–8 ng/mL, we anticipate that TTR might demonstrate a better performance metric for cutoff discrimination comparable to that of IPV.

Several limitations should be considered when interpreting our findings. First, this was a single-center study, which may limit the generalizability of the results to other populations and healthcare settings. Second, the retrospective nature of the analysis may have introduced selection bias. Third, although we identified IPV and TTR as predictors of adverse outcomes, the underlying mechanisms (such as non-adherence, genetic, or pharmacologic factors) linking high IPV and low TTR to graft dysfunction remain unclear. Additionally, we did not separate brand names of Prograf and Advagraf in our analysis, which may have influenced variability measurements.

## Conclusion

High IPV and low TTR are both significant predictors of adverse renal outcomes following kidney transplantation. Among these measures, IPV demonstrated superior discriminatory performance for predicting long-term graft function and BPAR in our clinical setting. Routine assessment of IPV in kidney transplant recipients may aid in early risk stratification and improve patient management, ultimately enhancing graft survival and overall outcomes.

## Supplementary Material

supplementary_data_Clean.docx
