# Supplementary material for: The predictive value of tacrolimus intrapatient variability and time in therapeutic range for renal transplant outcomes
Source: Ren Fail. 2025 Sep 3;47(1):2549395. doi: 10.1080/0886022X.2025.2549395 (PMC12409856; doi:10.1080/0886022X.2025.2549395)
Supplement: supplementary_data_Clean.docx [file IRNF_A_2549395_SM6586.docx]

Supplementary

Table 1. Baseline Characteristics of Patients Stratified by IPV (≥ 25.6% vs. < 25.6%) and TTR (≥ 81.1% vs. < 81.1%)

|  | Total | | IPV≥25.6+TTR<81.1 | | IPV≥25.6+TTR≥81.1 | | IPV<25.6+TTR<81.1 | | IPV<25.6+TTR≥81.1 | | *p* value |
| --- | --- | --- | --- | --- | --- | --- | --- | --- | --- | --- | --- |
| Patient number | 463 | | 129 | | 75 | | 65 | | 194 | |  |
| Kidney Transplant Recipient Age (years) | 47.3 | (36.8-54.9) | 47.6 | (38.0-55.3) | 47.4 | (36.7-56.3) | 47.6 | (41.1-52.7) | 46.2 | (35.4-55.2) | 0.674 |
| Gender |  |  |  |  |  |  |  |  |  |  | 0.844 |
| Female | 204 | (44.1%) | 61 | (47.3%) | 33 | (44.0%) | 28 | (43.1%) | 82 | (42.3%) |  |
| Male | 259 | (55.9%) | 68 | (52.7%) | 42 | (56.0%) | 37 | (56.9%) | 112 | (57.7%) |  |
| Donor type |  |  |  |  |  |  |  |  |  |  | 0.026* |
| Living donor | 165 | (35.6%) | 33 | (25.6%) | 29 | (38.7%) | 22 | (33.8%) | 81 | (41.8%) |  |
| Cadaveric donor | 298 | (64.4%) | 96 | (74.4%) | 46 | (61.3%) | 43 | (66.2%) | 113 | (58.2%) |  |
| Mean trough level of tacroliumus (ng/ml) | 7.28 | 1.92 | 7.17 | 2.71 | 7.74 | 1.03 | 6.11 | 2.26 | 7.55 | 1.22 | <0.001** |
| Median trough level of tacroliumus (ng/ml) (IQR) | 7.2 | (6.1-8.4) | 6.4 | (5.0-9.1) | 7.7 | (7.1-8.3) | 5.7 | (4.7-7.0) | 7.3 | (6.5-8.4) | <0.001** |
| IPV | 23.9% | (16.9%-33.7%) | 39.4% | (32.1%-50.8%) | 30.6% | (27.9%-36.9%) | 17.7% | (15.4%-22.2%) | 17.4% | (13.3%-20.7%) | <0.001** |
| TTR within 6-12 months after transplantation | 88.0% | (63.4%-100.0%) | 60.4% | (36.9%-72.4%) | 93.1% | (88.1%-97.3%) | 47.2% | (7.8%-72.5%) | 100.0% | (95.6%-100.0%) | <0.001** |
| TTR within 1 year after transplantation | 81.9% | (65.2%-92.2%) | 61.3% | (48.7%-75.5%) | 86.3% | (78.8%-93.1%) | 63.8% | (35.9%-79.0%) | 91.1% | (85.0%-95.1%) | <0.001** |
| Comorbidity |  |  |  |  |  |  |  |  |  |  |  |
| Pre-existing Diabetes Mellitus | 62 | (14.7%) | 20 | (17.7%) | 11 | (16.2%) | 11 | (18.0%) | 20 | (11.0%) | 0.334 |
| Pre-existing Hypertension | 295 | (63.7%) | 76 | (58.9%) | 40 | (53.3%) | 39 | (60.0%) | 140 | (72.2%) | 0.011* |
| Pre-existing Hepatitis C Virus | 20 | (4.5%) | 6 | (4.8%) | 5 | (7.6%) | 3 | (5.1%) | 6 | (3.1%) | 0.507 |
| Pre-existing Hepatitis B Virus | 38 | (8.6%) | 11 | (8.7%) | 5 | (7.5%) | 5 | (8.5%) | 17 | (8.9%) | 0.986 |
| Panel Reactive Antibody Test 1 | 42 | (9.1%) | 18 | (14.0%) | 4 | (5.3%) | 5 | (7.7%) | 15 | (7.8%) | 0.139 |
| Panel Reactive Antibody Test 2 | 27 | (5.9%) | 8 | (6.2%) | 7 | (9.3%) | 5 | (7.7%) | 7 | (3.6%) | 0.287 |
| Number of HLA Mismatches | 1.0 | (0.0-2.0) | 0.0 | (0.0-2.0) | 0.0 | (0.0-2.0) | 1.0 | (0.0-3.0) | 1.0 | (0.0-3.0) | 0.184 |
| ABO Incompatibility | 12 | (2.6%) | 3 | (2.3%) | 3 | (4.0%) | 1 | (1.5%) | 5 | (2.6%) | 0.823 |
| Use of Interleukin-2 Receptor Antibody for Induction | 155 | (34.6%) | 39 | (31.5%) | 19 | (26.0%) | 23 | (35.9%) | 74 | (39.6%) | 0.171 |
| Doubling of Serum Creatinine within 6–12 months | 50 | (10.8%) | 23 | (17.8%) | 8 | (10.7%) | 6 | (9.2%) | 13 | (6.7%) | 0.017* |
| Doubling of Serum Creatinine within 5 years | 20 | (4.3%) | 13 | (10.1%) | 5 | (6.7%) | 1 | (1.5%) | 1 | (0.5%) | 0.009** |
| Biopsy-Proven Acute Rejection within 1 year | 61 | (13.2%) | 23 | (17.8%) | 12 | (16.0%) | 6 | (9.2%) | 20 | (10.3%) | 0.155 |
| Biopsy-Proven Acute Rejection within 6-12 months after transplantation | 13 | (2.8%) | 5 | (3.9%) | 3 | (4.0%) | 1 | (1.5%) | 4 | (2.1%) | 0.635 |
| Mortality | 65 | (14.0%) | 22 | (17.1%) | 10 | (13.3%) | 8 | (12.3%) | 25 | (12.9%) | 0.711 |
| Graft failure | 97 | (21.0%) | 39 | (30.2%) | 12 | (16.0%) | 16 | (24.6%) | 30 | (15.5%) | 0.008** |

*Chi*-square test or Kruskal-Wallis test, Median (IQR). *P<0.05, **P<0.01

Figure 1. Patient selection algorithm.

All patients ever took tacrolimus between 1999-2018 (n=1000)

Total 537

Other organ transplant (n=34), 2^nd^ kidney transplant (n=19), 3 tacrolimus data at least within 1 year (n=290), cyclosporin shift to tacrolimus (n=77), tacrolimus shift to cyclosporin (n=1),

mTOR inhibitor (n=14), ever in clinical trial (n=2), loss of follow-up (n-41), not initially followed in this hospital (n=49), others (n=10) (incomplete follow-up data, early death (<1 month), or withdrawal of consent)

Cohort (n=463) for further analysis
